# The complete mitochondrial genome of a parthenogenetic ant *Monomorium triviale* (Hymenoptera: Formicidae)

**DOI:** 10.1080/23802359.2021.1970632

**Published:** 2021-08-31

**Authors:** Naoto Idogawa, Chih-Chi Lee, Chin-Cheng Scotty Yang, Shigeto Dobata

**Affiliations:** a Laboratory of Insect Ecology, Graduate School of Agriculture, Kyoto University, Kyoto, Japan; b Department of Evolutionary and Environmental Biology, Institute of Evolution, University of Haifa, Haifa, Israel; c Department of Entomology, Virginia Polytechnic Institute and State University, Blacksburg, VA, USA; d Department of General Systems Studies, Graduate School of Arts and Sciences, The University of Tokyo, Tokyo, Japan

**Keywords:** Hymenoptera, Formicidae, thelytoky, *Monomorium*, mitogenome

## Abstract

*Monomorium* is one of the most species-rich yet taxonomically problematic ant genus. An East Asian species, *M. triviale* Wheeler, W.M., 1906, is reproduced by obligate thelytokous parthenogenesis and performs strict reproductive division of labor. We sequenced the *M. triviale* mitogenome using next-generation sequencing methods. The circular mitogenome of *M. triviale* was 16,290 bp in length, consisting of 13 protein-coding genes, two ribosomal RNA genes, 22 transfer RNAs, and a single non-coding region of 568 bp. The base composition was AT-biased (82%). Gene order rearrangements were detected and likely to be unique to the genus *Monomorium*. We announce the *M. triviale* mitogenome as additional genomic resources for elucidating phylogenetic and taxonomic problems of *Monomorium* and comparative genomics of parthenogenetic ant species.

In the hyperdiverse ant subfamily Myrmicinae, *Monomorium* Mayr, 1855 is one of the most species-rich genera with over 300 described species including several successful tramp species such as the flower ant *M. floricola* and *M. salomonis*, and the pharaoh ant *M. pharaonis* (Pontieri and Linksvayer 2019). However, recent studies (Ward 2015; Sparks 2019) suggest polyphyly of this genus, and genomic resources are therefore essential for resolving such a taxonomic issue.

An East Asian species, *M. triviale* Wheeler, W.M., 1906 is particularly of our interest as it reproduces by thelytokous parthenogenesis where virgin queens produce both workers and next-generation queens (Gotoh et al. 2012; Idogawa et al. 2021). To date, only partial mitochondrial DNA sequences have been reported, with all of which being identical among populations in Japan (Idogawa et al. 2021). Hence, a complete mitochondrial genome of this species can provide additional information for further analysis. Here, we present the first complete mitogenome for *M. triviale*.

A colony of *M. triviale* headed by a single queen was collected in Takaragaike Park, Kyoto, Japan (35.060087 N, 135.788488 E) on 9 September 2017. The queen and her parthenogenetic offspring produced later in the laboratory (larvae and pupae, approximately 100 individuals) were fixed in 99.5% EtOH. We extracted genomic DNA from the pooled individuals with DNeasy Blood and Tissue kit (Qiagen, Hilden, Germany). We sequenced the pooled DNA on the HiSeq X sequencer (Illumina, San Diego, CA) at Macrogen Japan Corp. (Tokyo, Japan). After removing adapters with Trimmomatic v0.39 (Bolger et al. 2014), we conducted de novo mitogenome assembly based on 4,327,159 paired-end sequence reads using NOVOPlasty v3.6 (Dierckxsens et al. 2017), with *Solenopsis invicta* mitogenome (NCBI accession: NC_014672) as a seed. Average read coverage of the mitogenome assembly was 37,840, providing ample depth for correctness. We annotated protein-coding genes (PCGs), rRNAs, and tRNAs using GeSeq (Tillich et al. 2017), MITOS (Bernt et al. 2013), NCBI ORF-finder (Rombel et al. 2002), and ARWEN (Laslett and Canbäck 2008). The sequence information was deposited in the DNA Data Bank of Japan under accession number: LC605004. A specimen was deposited at the Laboratory of Insect Ecology, Graduate School of Agriculture, Kyoto University (http://www.insecteco.kais.kyoto-u.ac.jp; N. Idogawa; under the voucher number Mtri_20170909_4).

The complete mitogenome of *M. triviale* was 16,920 bp in length, which is comparable to those of other ant species. The nucleotide composition was AT-biased (82%). The mitogenome contained 13 PCGs, two rRNAs, and 22 tRNAs, which are typical for most metazoan animals. All PCGs used ATG or ATT as the start codon and TAA or TAG as the stop codon. The tRNAs, ranging in size from 59 to 74 bp, were similar to those of other ants (54–90 bp). The control region presumably corresponded to the single largest non-coding AT-rich region (569 bp, A + T 94%). Gene order of *M. triviale* was identical to that of a congener *M. pharaonis* (NC_051486.1). Notably, the gene order of the two *Monomorium* species had two gene rearrangements: an inversion between *trn*P and *ND1* (Myrmicinae, *ND6*-*CYTB*-*trn*S; *Monomorium*, *trn*S-*CYTB*-*ND6*; underlines indicate inverted genes) and translocations between *ND3* and *trn*F (Myrmicinae common, *trn*A-*trn*R-*trn*N-*trn*S-*trn*E; *Monomorium*, *trn*R-*trn*E-*trn*A-*trn*N-*trn*S). This feature was different from the common gene order of the subfamily Myrmicinae and likely unique to *Monomorium* ants (Babbucci et al. 2014; Park 2020). This may help identify the genus *Monomorium sensu stricto*, in addition to nucleotide substitutions.

We inferred the phylogenetic relationships of 25 ant species using the concatenated nucleotide sequences of all 13 PCGs, with the honeybee *Apis mellifera* as an outgroup. Sequence alignment was constructed using ClustalW (Thompson et al. 2003) implemented in MEGA-X (Kumar et al. 2018). The GTR + I + G model was determined as a best-fit model by ModelTest-NG v0.1.6 (Darriba et al. 2020). Both a maximum-likelihood tree made by RAxML-NG v1.0.0 (Kozlov et al. 2019) and Bayesian inference tree made by MrBayes v3.2.7 (Ronquist et al. 2012) consistently support the current phylogenetic placement of *Monomorium* in the subfamily Myrmicinae (Figure 1).

**Figure 1.**
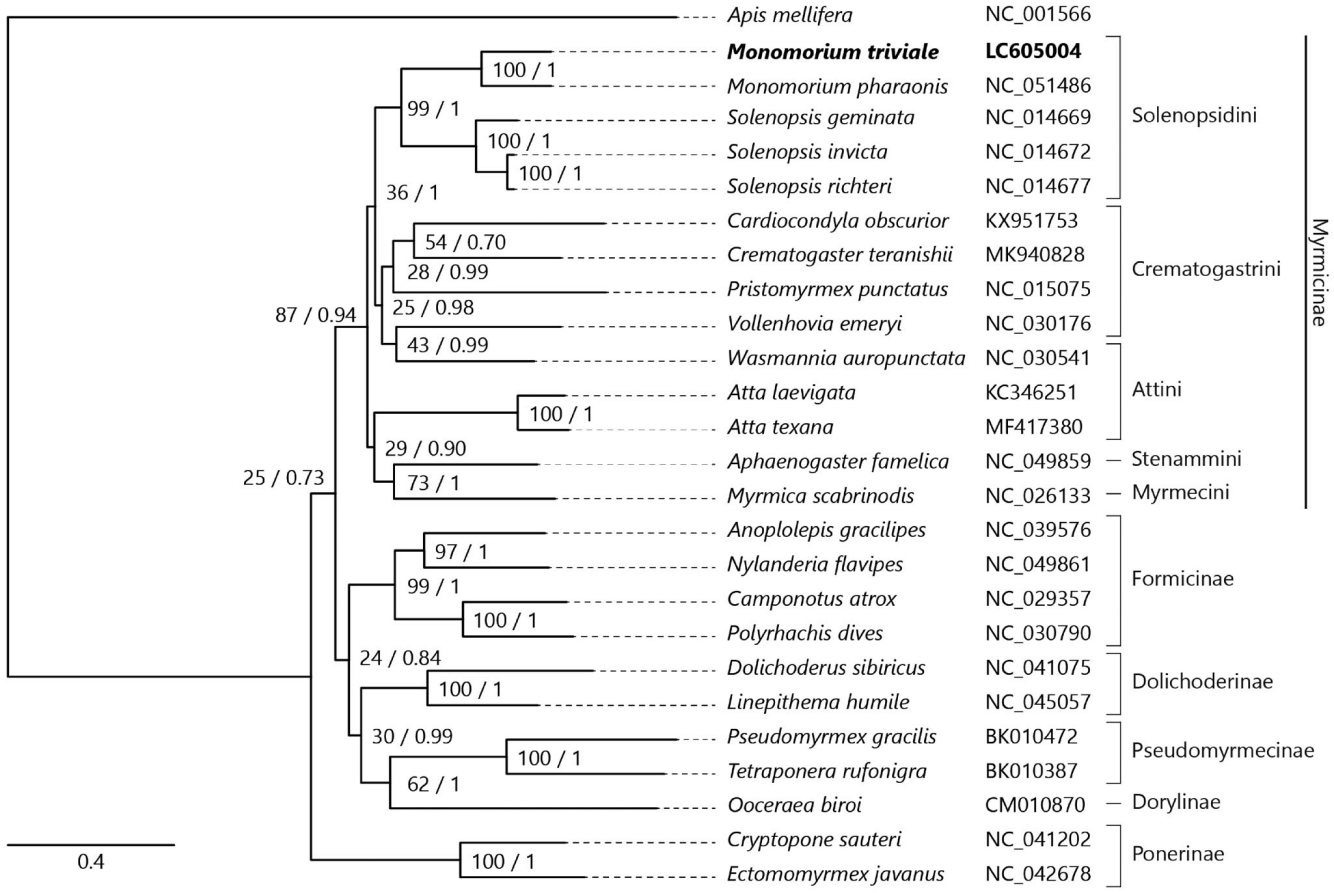
Maximum-likelihood (1000 bootstrap repeats) and Bayesian inference (100,000 generations) trees showing phylogenetic relationships among 25 ant species. A honeybee, *Apis mellifera* was used as an outgroup. Phylogenetic tree was drawn based on maximum-likelihood tree. *Monomorium triviale* in bold is the result obtained in this study. The numbers above branches indicate bootstrap support values for maximum-likelihood tree and posterior probability for Bayesian inference tree, in order.

In conclusion, the newly sequenced complete mitochondrial genome of *M. triviale* provides additional resources for further phylogenetic characterization of the taxonomically problematic genus *Monomorium* and comparative genomics of parthenogenetic ant species.

## Data Availability

The genome sequence data that support the findings of this study are openly available in DDBJ/GenBank at https://www.ddbj.nig.ac.jp under the accession no. LC605004. The associated BioProject, SRA, and Bio-Sample numbers are PRJDB12079, DRX301164, and SAMD00394597, respectively.

## References

[CIT0001] Babbucci M, Basso A, Scupola A, Patarnello T, Negrisolo E. 2014. Is it an ant or a butterfly? Convergent evolution in the mitochondrial gene order of Hymenoptera and Lepidoptera. Genome Biol Evol. 6(12):3326–3343. 25480682 10.1093/gbe/evu265 PMC4466343

[CIT0002] Bernt M, Donath A, Jühling F, Externbrink F, Florentz C, Fritzsch G, Pütz J, Middendorf M, Stadler PF. 2013. MITOS: improved de novo metazoan mitochondrial genome annotation. Mol Phylogenet Evol. 69(2):313–319. 22982435 10.1016/j.ympev.2012.08.023

[CIT0003] Bolger AM, Lohse M, Usadel B. 2014. Trimmomatic: a flexible trimmer for Illumina sequence data. Bioinformatics. 30(15):2114–2120. 24695404 10.1093/bioinformatics/btu170 PMC4103590

[CIT0004] Darriba D, Posada D, Kozlov AM, Stamatakis A, Morel B, Flouri T. 2020. ModelTest-NG: a new and scalable tool for the selection of DNA and protein evolutionary models. Mol Biol Evol. 37(1):291–294. 31432070 10.1093/molbev/msz189 PMC6984357

[CIT0005] Dierckxsens N, Mardulyn P, Smits G. 2017. NOVOPlasty: de novo assembly of organelle genomes from whole genome data. Nucleic Acids Res. 45(4):e18. 28204566 10.1093/nar/gkw955 PMC5389512

[CIT0006] Gotoh A, Billen J, Tsuji K, Sasaki T, Ito F. 2012. Histological study of the spermatheca in three thelytokous parthenogenetic ant species, *Pristomyrmex punctatus*, *Pyramica membranifera* and *Monomorium triviale* (Hymenoptera: Formicidae). Acta Zool. 93(2):200–207.

[CIT0007] Idogawa N, Sasaki T, Tsuji K, Dobata S. 2021. Comprehensive analysis of male-free reproduction in *Monomorium triviale* (Formicidae: Myrmicinae). PLOS One. 16(4):e0246710. 33914749 10.1371/journal.pone.0246710 PMC8084239

[CIT0008] Kozlov AM, Darriba D, Flouri T, Morel B, Stamatakis A. 2019. RAxML-NG: a fast, scalable and user-friendly tool for maximum likelihood phylogenetic inference. Bioinformatics. 35(21):4453–4455. 31070718 10.1093/bioinformatics/btz305 PMC6821337

[CIT0009] Kumar S, Stecher G, Li M, Knyaz C, Tamura K. 2018. MEGA X: molecular evolutionary genetics analysis across computing platforms. Mol Biol Evol. 35(6):1547–1549. 29722887 10.1093/molbev/msy096 PMC5967553

[CIT0010] Laslett D, Canbäck B. 2008. ARWEN: a program to detect tRNA genes in metazoan mitochondrial nucleotide sequences. Bioinformatics. 24(2):172–175. 18033792 10.1093/bioinformatics/btm573

[CIT0011] Park J, Xi H, Park J. 2020. The complete mitochondrial genome of *Aphaenogaster famelica* (Smith, 1874) (Hymenoptera: Formicidae). Mitochondrial DNA B Resour. 5(1):492–494. 33366617 10.1080/23802359.2019.1698992 PMC7748472

[CIT0012] Pontieri L, Linksvayer TA. 2019. Monomorium. In: Starr C. (ed.), Encyclopedia of social insects. Cham: Springer International Publishing; p. 1–6.

[CIT0013] Rombel IT, Sykes KF, Rayner S, Johnston SA. 2002. ORF-FINDER: a vector for high-throughput gene identification. Gene. 282(1–2):33–41. 11814675 10.1016/s0378-1119(01)00819-8

[CIT0014] Ronquist F, Teslenko M, van der Mark P, Ayres DL, Darling A, Höhna S, Larget B, Liu L, Suchard MA, Huelsenbeck JP, et al. 2012. MrBayes 3.2: efficient Bayesian phylogenetic inference and model choice across a large model space. Syst Biol. 61(3):539–542. 22357727 10.1093/sysbio/sys029 PMC3329765

[CIT0015] Sparks KS, Andersen AN, Austin AD. 2019. A multi-gene phylogeny of Australian *Monomorium* Mayr (Hymenoptera: Formicidae) results in reinterpretation of the genus and resurrection of *Chelaner* Emery. Invertebr Syst. 33:225–236.

[CIT0016] Thompson JD, Gibson TJ, Higgins DG. 2003. Multiple sequence alignment using ClustalW and ClustalX. Curr Protoc Bioinformatics. 10.1002/0471250953.bi0203s00 18792934

[CIT0017] Tillich M, Lehwark P, Pellizzer T, Ulbricht-Jones ES, Fischer A, Bock R, Greiner S. 2017. GeSeq- versatile and accurate annotation of organelle genomes. Nucleic Acids Res. 45(W1):W6–W11. 28486635 10.1093/nar/gkx391 PMC5570176

[CIT0018] Ward PS, Brady SG, Fisher BL, Schultz TR. 2015. The evolution of myrmicine ants: phylogeny and biogeography of a hyperdiverse ant clade (Hymenoptera: Formicidae). Syst Entomol. 40(1):61–81.

